# What is the recommended management of a young woman with an intact endometrioma desiring future fertility?

**DOI:** 10.3389/fendo.2022.1005597

**Published:** 2022-11-10

**Authors:** Johnny S. Younis, Scott M. Nelson

**Affiliations:** ^1^ Reproductive Medicine, Department of Obstetrics and Gynecology, Baruch-Padeh Medical Center, Poriya, Israel; ^2^ Azrieili Faculty of Medicine, Bar-Ilan University, Safed, Israel; ^3^ School of Medicine, Glasgow Royal Infirmary, University of Glasgow, Glasgow, United Kingdom; ^4^ National Institute of Health Research (NIHR) Bristol Biomedical Research Centre, Bristol, United Kingdom; ^5^ The Fertility Partnership, Oxford, United Kingdom

**Keywords:** endometrioma, endometriotic cystectomy, ovarian reserve, clinical pregnancy rate, live-birth rate, anti-Müllerian hormone, antral follicle count

## Introduction

Endometriosis is a common, chronic, and inflammatory illness, with endometrioma a distinct advanced and progressive manifestation of the disease associated with reproductive dysfunction, infertility, and the need for ART treatment. Endometrioma is the most frequently diagnosed form of the disease, identified in up to 44% of affected women. However, the temporal management of endometriomas in women wishing to conceive or relative to ART treatment is not standardized, with surgery frequently advised pre-conceptually and before IVF treatment (1, 2). Despite recent guidelines suggesting that clinicians may consider laparoscopy to treat infertility, routine endometrioma removal before ART is not indicated (3). Reevaluation of this practice within a critical framework that addresses the impact of endometrioma on ovarian reserve and whether its removal is beneficial in terms of fecundity and efficacy of ART treatment is, therefore, timely. This commentary explores recent evidence in a pragmatic question-driven approach and proposes a suggested clinical management strategy.

## Does an endometrioma per se affect ovarian reserve?

This is one of the most controversial topics related to the pathophysiology of endometrioma in the biology of ovarian reserve. Several mechanisms are suggested to expound on the adverse effect of endometrioma per se on early folliculogenesis and ovarian reserve. Firstly, the mere mechanical stretching effect of the endometrioma on surrounding cortical tissue (4). Secondly, an endometrioma contains various toxic elements that have the aptitude to affect the adjacent normal cortical ovarian tissue. In high concentrations, these toxic elements contain free iron radicals, reactive oxygen species, proteolytic enzymes, and inflammatory molecules. These elements, mediated by macrophages, cytokines, and vasoactive substances, may cause a hostile environment causing inflammation and fibrosis (5). Thirdly, endometrioma triggering a local pelvic inflammation may show activated follicular recruitment and atresia of early follicles, leading to a “burnout” effect (6).

Nevertheless, although there are some clues for molecular, histological, and morphological mechanisms that may back up these mechanisms, clarifying the negative impact of endometrioma on the adjacent ovarian cortical tissue and early folliculogenesis, the evidence is far from conclusive (7). Further investigation at the molecular and cellular level ought to be invested in clarifying the magnitude of endometriotic gonadotoxic insult to the ovarian reserve.

The topic is also still debatable in the clinical setting. Pros and cons of the adverse effect of endometrioma on the functional ovarian reserve are available (8, 9). However, these studies typically include a small number of women. In one prospective study, median serum AMH levels declined by 26.4% six months apart in women with an intact endometrioma compared to 7.4% in controls (9). Conversely, in another retrospective study, ovarian responsiveness to controlled ovarian stimulation did not differ, at least six months apart, between women with an intact endometrioma and others with normal ovaries. Larger series and more extended periods of follow-up are required for definite conclusions.

A recently published systematic review and meta-analysis that targeted this question included 17 studies comprising 968 and 1874 women, with and without endometrioma, respectively. About 30% of studies (35% of women) had a retrospective design (10). Serum anti-Müllerian hormone (AMH) levels were reduced in patients with ovarian endometriomas compared to women with either healthy ovaries or other benign ovarian cysts, equivalent to -0.84 ng/mL (95% CI: -1.16 to -0.52), suggesting a damaging effect of the endometrioma per se to the ovarian reserve.

Conversely, a more recent systematic review and meta-analysis explored endometrioma laterality (uni- versus bilateral) as a surrogate for disease severity on ovarian reserve (11). Twelve studies, all prospective in design, were eligible for meta-analysis and collectively included 783 women: 489 unilateral and 294 bilateral. The pre-operative weighted mean difference showed that serum AMH levels did not differ significantly between unilateral or bilateral disease women. This suggests that despite bilateral disease representing a more progressive and advanced disease, there was no adverse impact on the ovarian reserve, challenging the concept that an intact endometrioma reduces the functional ovarian reserve.

## What is the impact of endometrioma surgery on ovarian reserve?

Histological studies revealed that endometriotic cystectomy is commonly complicated by the inadvertent removal of normal ovarian follicles adjacent to the pseudo-capsule, which seems unavoidable even in the hands of experienced surgeons (12–14). This iatrogenic damage may result from inevitable manipulation of the cortex with tearing of tissue planes and even minimal bleeding associated with coagulation damage.

Endometriotic stripping cystectomy has also been shown to have an adverse impact on serum AMH levels (15). In a recent systematic review and meta-analysis, the weighted mean difference (WMD) of serum AMH levels dropped significantly by 1.65 ng/ml (95% CI: 1.15 to 2.15) and by 2.03 ng/mL (95% CI: 1.47 to 2.58) at 9-12 months postoperatively as compared to basal levels in the unilateral and bilateral endometriotic cystectomy groups, respectively, equivalent to 39% and 57% decrease following the operation (11).

In this regard, several systematic reviews and meta-analyses have examined whether different hemostasis means during endometriotic cystectomy may have an altered impact on ovarian reserve (16–18). In all of these studies, bipolar coagulation was more detrimental than other non-thermal methods (sutures or hemostatic sealants). In one meta-analysis, the mean decline in serum AMH levels was about 7% less with non-thermal methods than with bipolar coagulation (16). Notably, these meta-analyses included retrospective studies, a modest number of women (n = 105-312), and evaluated serum AMH levels only once, three months following surgery.

Collectively, this histological and functional data would suggest that endometrioma cystectomy has an immediate impact on the ovarian reserve, which is clinically detectable by AMH. Bipolar coagulation should be cautiously limited in these cases.

## Is the impact of endometriotic cystectomy on ovarian reserve reversible?

That histology studies show loss of regular primordial follicles adjacent to the removed endometrioma specimens suggests that the impact on the ovarian reserve is not reversible (12–14).

Furthermore, two recent systematic reviews and meta-analyses of prospective studies reported that AMH concentrations remained irrevocably reduced by an estimated 40-53% at 9-18 months postoperatively (11, 19), consistent with a permanent impact of ovarian cystectomy on ovarian reserve and reproductive lifespan.

## Could endometriotic cystectomy lead to premature ovarian insufficiency?

Although reported in both the immediate and late postsurgical period (20–22) and associated with an increased risk of earlier menopause (21), POI is an uncommon complication affecting up to 2.4% of women after endometriotic cystectomy (20). POI primarily developed in women having bilateral endometriotic cystectomy or in conjugation with repeat surgery (20, 22). This risk relationship was further modified by age, with older women at the time of surgery having a greater risk of POI (correlation coefficient: -0.63, Spearman’s correlation coefficient by rank test) (22).

## Which ovarian reserve test, AMH or AFC, is more reliable in cases with endometrioma?

Previous systematic reviews and meta-analyses have either evaluated antral follicle count (AFC) or AMH with discordant outcomes and confusing clinical messages (15, 23). A recent meta-analysis undertook a different methodology to overcome potential measurable and non-measurable confounders, evaluating repeat, concomitant, and parallel measures of AMH and AFC in the same women, settings, and periods (19). Fourteen prospective studies and 650 women were included. Endometriotic cystectomy in the pooled prospective studies was associated with a significant reduction in serum AMH but not AFC, with detrimental effects consistently detectable for AMH at the early, intermediate, and late postoperative time intervals corresponding to 1.77 ng/mL (95% CI: 0.77 to 2.77), 1.17 ng/mL (95% CI: 0.66 to 1.67) and 2.13 ng/mL (95% CI: 1.61 to 2.65), respectively. In contrast, AFC estimates did not change significantly in the parallel periods despite being simultaneously measured in the same women. These results suggest that AMH is a more sensitive biomarker of ovarian reserve than AFC and should be routinely incorporated in women’s pre and post-operative counseling considering endometriotic surgery.

## Does an endometrioma cystectomy improve IVF results?

Several systematic reviews and meta-analyses have consistently found that the number of retrieved oocytes, clinical pregnancy, and live birth rates were comparable in women following endometriotic cystectomy compared to controls with intact endometrioma (24–27).

The most cited is that of Hamdan et al. (25), which included 33 studies, mostly retrospective in design. Compared with women with no surgical intervention, women who underwent endometriotic cystectomy before IVF had a similar clinical pregnancy rate (OR 0.97; 95% CI: 0.78 to 1.2), containing 11 studies and 1521 women, and a similar live birth rate (OR 0.90; 95% CI: 0.63 to 1.28), analyzing five studies and 655 women.

This suggests that it should not be undertaken routinely; however, cystectomy may be appropriate to improve the accessibility of follicles or to improve quality of life measures while undertaking assisted conception (3).

## Does an intact endometrioma reduce the chance of pregnancy in IVF?

Several systematic reviews and meta-analyses were published targeting IVF results in women with intact endometrioma compared to controls with normal ovaries or no endometrioma (16, 19, 20). Although the number of retrieved oocytes was reduced in women with an intact endometrioma, clinical pregnancy and live birth rates were comparable to controls, negating an adverse effect of an intact endometrioma on pregnancy attainment.

In the study of Hamdan et al (25), compared with women with no endometrioma undergoing IVF, women with an intact endometrioma had a similar clinical pregnancy rate (OR1.17; 95% CI: 0.87 to 1.58) and a similar live birth rate (OR 0.98; 95% CI 0.71 to 1.36), analyzing five studies and 928 women.

It is important to emphasize that all published meta-analyses targeting ART results in women with endometrioma included mainly retrospectively designed studies (24–29), which may hamper the estimates and evidence obtained. Further prospective well-controlled studies are needed for confirmation of these findings.

## Does conservative management increase the risk of endometrioma complications during ART?

The risk of complications that an intact endometrioma poses during ART include abnormal oocyte competence, technical difficulties during oocyte retrieval, endometrioma rupture, injury to adjacent organs, infection, follicular fluid contamination with endometrioma content, and pregnancy complications. Although all risks may be theoretically reduced with cystectomy, the most expected risk without surgery was an infection, with an incidence of 0.6% (30); hence, routine antibiotic administration should be considered at the time of oocyte retrieval (3).

## Does ovarian stimulation before ART increase the risk of endometriosis progression?

Low-quality evidence suggests deep invasive endometriosis may progress with controlled ovarian stimulation (31). In contrast, there is moderate evidence to suggest that IVF-ET does not worsen pain related to endometriosis nor increase the risk of endometriosis recurrence (31).

## Is there an increased risk of ovarian cancer occurrence in an endometrioma?

The likelihood of developing ovarian cancer within an endometrioma is rare at reproductive age. The lifetime ovarian cancer risk in the general population is estimated to be 1.31% compared to 1.80% in women with endometriosis, with a low relative risk of 1.42% (32). In a recent nationwide Dutch study of 131,240 women with histologically diagnosed endometriosis and 132,654 matched controls, a higher incidence of clear-cell, endometrioid, and all ovarian cancer subtypes was found in women with histologically proven endometriosis (33). However, in many of these women, endometriosis and ovarian cancer were diagnosed synchronously after the average menopausal age. This suggests that the risk of ovarian cancer in endometriosis patients remains, even when symptoms are no longer present. This would indicate that long-term follow-up is required.

## Conclusion

Collectively these data would support conservative management of endometrioma until reproductive aspirations are realized. Our recommendation for the avoidance of routine endometriotic cystectomy is consistent with international guidelines. It is based on multiple synergistic and interconnected queries directly related to the effect of ovarian endometrioma on ovarian reserve and the probability of attaining pregnancy. Conservative management should be encouraged even when ART is performed unless there is a considerable risk of endometrioma complications. We do, however, acknowledge the lack of evidence either way for surgery when ART has failed repeatedly.

Other situations where diagnostic laparoscopy should be considered include women with endometriosis-associated pelvic pain when medical therapy has failed. Surgical treatment should also be discussed in ART cases where developing follicles cannot be reached during oocyte retrieval. In addition, in patients with endometrioma developing manifestations of endometriosis-associated ovarian cancer, although rare in reproductive age, surgery and histologic evaluation may be inevitable for final diagnosis and treatment.

We appreciate that many of the presented studies have been retrospective. Future prospective studies will refine risk estimates further; until then, avoiding iatrogenic deleterious and sustained effects on the ovarian reserve with little upside should be avoided.

In addition, future studies should explore other advanced (non-conservative) modalities of endometrioma treatment impacting ovarian reserves, such as ultrasound-guided sclerotherapy or laser vaporization, compared to cystectomy to advise young women desiring future fertility properly. Furthermore, fertility preservation should be discussed in these women, especially when surgery is unavoidable and in cases where ovarian reserve is *a priori* impaired.

## Author contributions

JSY contributed to the conception and design of the manuscript and drafted the article. SN edited and revised the manuscript for important intellectual content. All authors contributed to the article and approved the submitted version.

## Conflict of interest

SN reports personal fees from Access Fertility, personal fees from Merck, personal fees from Ferring, grants and personal fees from Roche Diagnostics, personal fees from The Fertility Partnership, and personal fees from Modern Fertility, outside the submitted work.

The remaining author declares that the research was conducted in the absence of any commercial or financial relationships that could be construed as a potential conflict of interest.

## Publisher’s note

All claims expressed in this article are solely those of the authors and do not necessarily represent those of their affiliated organizations, or those of the publisher, the editors and the reviewers. Any product that may be evaluated in this article, or claim that may be made by its manufacturer, is not guaranteed or endorsed by the publisher.
